# Insomnia and Its Association with Successful Aging in the Older Indian Population: A Large Population-Based Study Based on LASI, Wave 1

**DOI:** 10.1192/j.eurpsy.2026.11989

**Published:** 2026-07-31

**Authors:** S. Leng, Y. Jin, X. Tang

**Affiliations:** West China Hospital, Sichuan University, Chengdu, China

## Abstract

**Introduction:**

Evidence regarding the link between insomnia and successful aging (SA) in the older generation remains scarce.

**Objectives:**

The purpose of this study is to explore the relationship of insomnia with SA within a substantial sample of the community-dwelling Indian population.

**Methods:**

Data were drawn from the Longitudinal Ageing Study in India (LASI), Wave 1, conducted during 2017-2018. Older participants aged 60 years and above who completed both the insomnia and SA surveys were included. Insomnia was determined by the presence of at least one of three symptoms: 1) difficulty in initiating sleep; 2) difficulty in maintaining sleep; or 3) early morning awakening, occurring 5 or more times per week. SA was assessed by five components: 1) absence of chronic diseases; 2) low probability of disability; 3) high cognitive functionality; 4) low probability of depression; and 5) active social engagement. The association between insomnia and SA was examined through survey-weighted multivariable logistic regression, with adjustments made for potential covariates. Subgroup analyses were carried out to evaluate interactions with age, sex, alcohol use, and smoking status.

**Results:**

A total of 31362 participants met the eligibility criteria. The overall weighted prevalence was 9.91% for insomnia and 23.94% for SA. In fully adjusted models, insomnia exhibited a negative association with SA (OR 0.70; 95% CI 0.63-0.78, see Table 1) and with each of SA’s components, except for the absence of chronic diseases (OR 0.94; 95% CI 0.85-1.04, see Table 1). Subgroup analyses, stratified by age, sex, alcohol use, or smoking status, did not reveal any significant interactions between insomnia and SA (p for interaction = 0.098, 0.873, 0.704, 0.095, respectively; refer to Table 2).

**Conclusions:**

Insomnia was negatively linked with SA within the older Indian population. Future prospective studies are warranted to validate these relationships, investigate underlying mechanisms, and enhance the understanding and promotion of SA.

**Disclosure of Interest:**

None Declared

